# Epigenomics in Hurthle Cell Neoplasms: Filling in the Gaps Towards Clinical Application

**DOI:** 10.3389/fendo.2021.674666

**Published:** 2021-05-24

**Authors:** Sule Canberk, Ana Rita Lima, Mafalda Pinto, Paula Soares, Valdemar Máximo

**Affiliations:** ^1^ Instituto de Investigação e Inovação em Saúde (i3S), University of Porto, Porto, Portugal; ^2^ Cancer Signaling and Metabolism Group, Institute of Molecular Pathology and Immunology of the University of Porto (Ipatimup), Porto, Portugal; ^3^ Abel Salazar Institute of Biomedical Sciences (ICBAS), University of Porto, Porto, Portugal; ^4^ Faculty of Medicine, University of Porto (FMUP), Porto, Portugal; ^5^ Department of Pathology, Faculty of Medicine, University of Porto, Porto, Portugal

**Keywords:** thyroid tumors, Hürthle cells, oncocytic cells, mitochondria, epigenomics, epigenetics analysis, Hürthle cell tumors, Hürthle cell carcinoma

## Abstract

It has been widely described that cancer genomes have frequent alterations to the epigenome, including epigenetic silencing of various tumor suppressor genes with functions in almost all cancer-relevant signalling pathways, such as apoptosis, cell proliferation, cell migration and DNA repair. Epigenetic alterations comprise DNA methylation, histone modification, and microRNAs dysregulated expression and they play a significant role in the differentiation and proliferation properties of TC. In this review, our group assessed the published evidence on the tumorigenic role of epigenomics in Hurthle cell neoplasms (HCN), highlighting the yet limited, heteregeneous and non-validated data preventing its current use in clinical practice, despite the well developed assessment techniques available. The identified evidence gaps call for a joint endeavour by the medical community towards a deeper and more systematic study of HCN, aiming at defining epigenetic markers in early diagnose, allowing for accurate stratification of maligancy and disease risk and for effective systemic treatment.

## Introduction

### What are Hürthle Cell Tumors?

Only a few neoplasms have been gaining so many names in their history as oncocytic thyroid neoplasms, reflecting the uncertainty about their nature: Askanazy cell tumor, oxyphilic cell tumor, Langhan’s Struma, Baber cell tumor, Getzowa’s Struma, Hürthle cell tumor, oncocytic cell tumor, among others (1). In 2004, the 3^th^ edition of the World Health Organization (WHO) Classification of Tumors endorsed the term “oncocytic tumors”, instead of “Hürthle cell tumors”, to clarify that 75% of oncocytes were classified as a variant under the category of papillary thyroid carcinoma (PTC) and follicular thyroid carcinoma (FTC) (2). The 4^th^ edition of the WHO Classification of Tumors establishes a new tumor entity under the title of Hürthle cell neoplasms (HCN) (3). The unifying feature in those tumors is the cytoplasmatic enrichement in mithocondria (from several hundreds to thousands), that gives the characteristic acidophilic, granular aspect to oncocytes.

Hürthle cell neoplasms (HCN) are rare in comparison with non-oncocytic follicular cell-derived neoplasms, with variable incidence and prevalence in different studies. According to the 4^th^ edition of the WHO Classification of Tumors (3), there are two types of HCN: Hürthle cell adenoma (HCA) and Hurthel cell carcinoma (HCA), both composed by 75% or more of Hürthle cells (2). The presence or absence of invasion into the tumor capsule and vessel (angio) distinguishes HCA from HCC. Similar to FTCs, HCCs are classified as minimally invasive, encapsulated angioinvasive and widely invasive (2).

HCCs are referred to as poorly radioiodine avid and poorly responsive to chemotherapy and radiation. Outcomes of patients with widely invasive HCC are poorer compared with patients with minimally invasive HCC (3–6). Despite their unique morphology and molecular background, no well-defined molecular markers exist in daily practice for HCC. This becomes more of an unmet need not only in the diagnose, but also for the treatment of these tumors. Indeed, HCC corresponds to 3% to 7% of all differentiated thyroid cancers, but ranges from 10.5% to 43% in recurrent cases, which highlights the unmet need for effective treatment strategies (4–7).

### What Is Epigenetics?

Epigenetics means “beyond genetics,” and it represents diverse mechanisms that modify both gene expression and genome stability without affecting the DNA sequence itself. The term “epigenetics” was first coined by Waddington in the early 1940s (8). He described the term as “the causal interactions between genes and their products, which bring the phenotype into being” (8). After many advances achieved over decades, this eloquent description still faithfully describes the main characteristic of epigenetics: phenotype changes in gene expression, which are not related with changes in the DNA sequence both in mitosis and between generations. The effects of epigenetical mechanisms on DNA are not “additional” or “contrary” to genetical mechanisms, but are manifested by an interplay between genetic and epigenetic mechanisms on the roadmap to cancer transformation.

**Figure 1:**
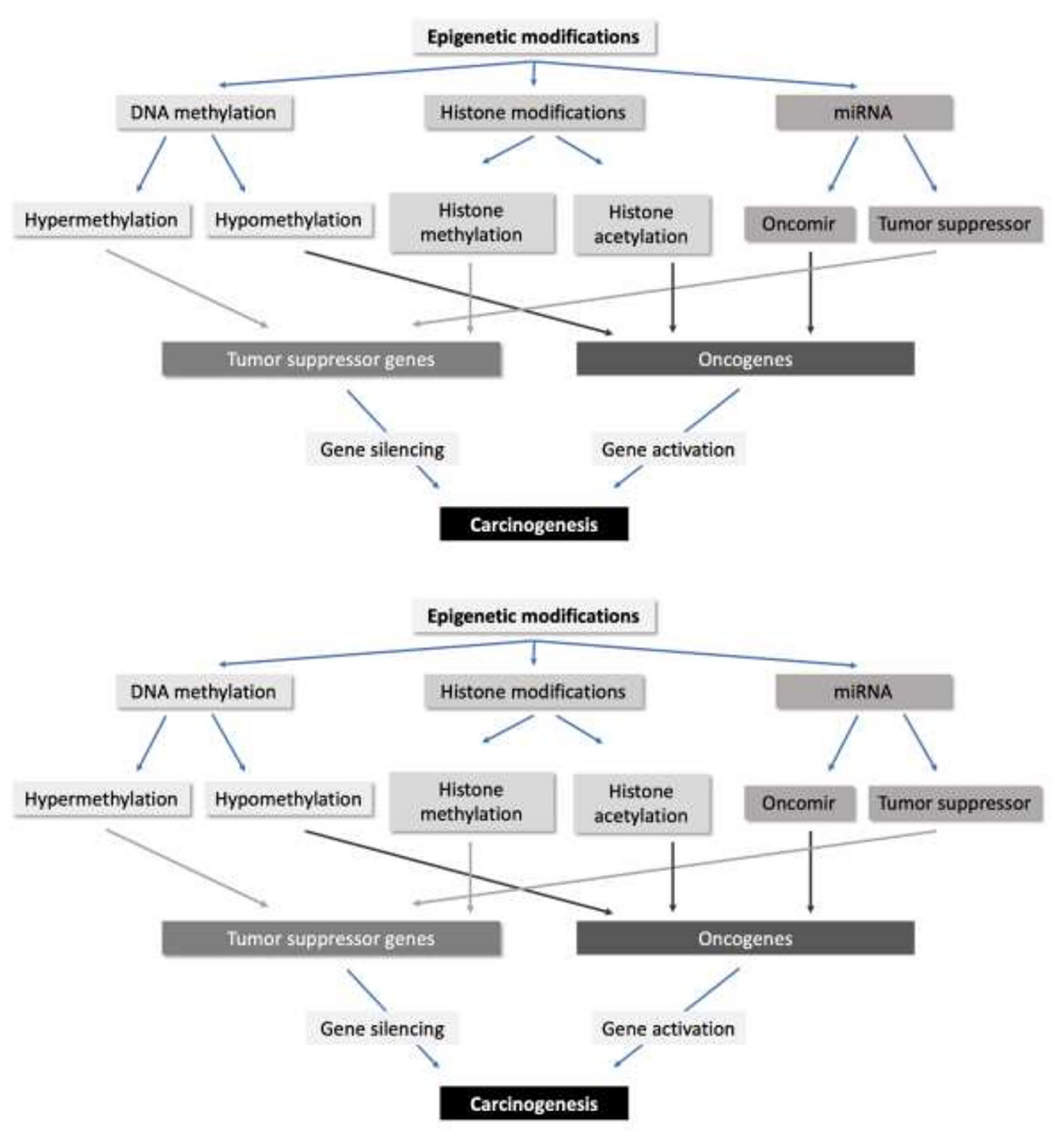
Overview of the epigenetic modifications in human cells and their influences in carcinogenesis.

Gene transcription strongly depends on the chromatin structure: in general, the “switch on” or open/decondensed (euchromatin) states are transcriptionally active, whereas the “switch off” or close/condensed (heterochromatin) states are transcriptionally inactive. This continuous interaction between the chromatin remodelling processes is currently denominated “epigenome” – the epigenetic status that determines the way a single eukaryotic genome may manifest it-self in different cell types and developmental stages and which, if aberrant, give rise to cancer and other diseases (9). These conformational changes of epigenetic regulation that may cause mitotically and/or meiotically heritable changes in gene function, without entailing any change in DNA sequence, can be classified into three distinct types: DNA methylation, histone modifications, and non-coding ribosomal RNAs (rRNAs) (**Figure 1**) (9–11). Each of these epigenetic pathways involve enzymes that transfer the modification (‘writers’), enzymes that modify or revert a modification (‘erasers’), and enzymes that mediate the interactions of proteins or protein complexes with the modification (‘readers’) (12). It is well-known that cancer genomes show frequent alterations of the epigenome, including epigenetic silencing of various tumor suppressor genes with functions in almost all cancer-relevant signalling pathways, such as apoptosis, cell proliferation, cell migration and DNA repair (12).

Epigenetic alterations also play a highly significant role in the differentiation and proliferation properties of thyroid carcinoma (TC) (11, 12). In this review, our group covers the role of epigenomics in the HCN, along with the main epigenetic mechanisms and potential therapy application of epigenetic targeted therapies in HCC.

### Molecular Techniques for the Analysis of Epigenetic Alterations

Epigenetic alterations, such as DNA methylation and histone modification, as well as microRNAs dysregulated expression, may serve as biomarkers for early detection, disease classification and risk stratification, as well as targets for therapy and chemoprevention.

**Figure 2:**
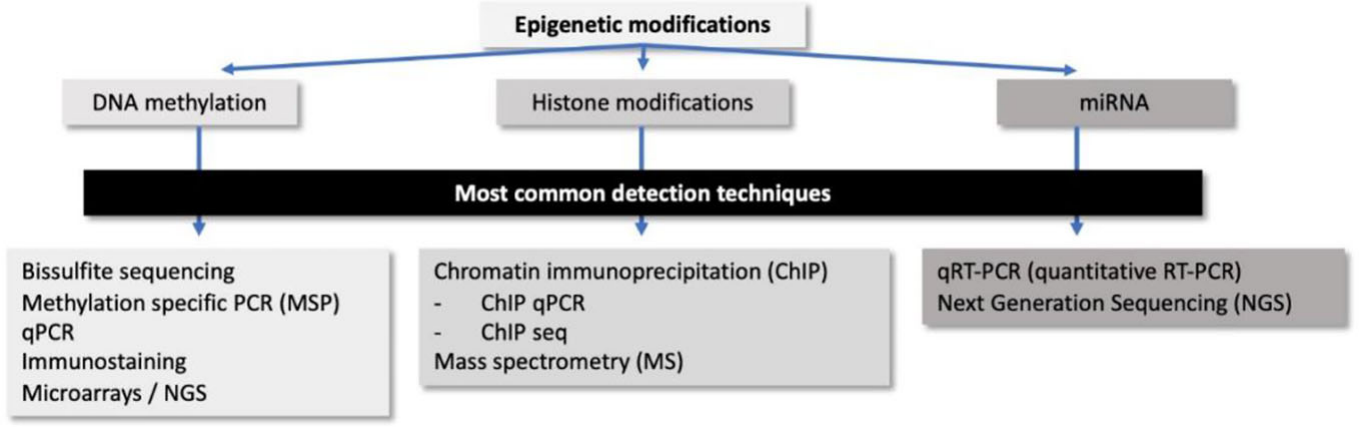
Summary of the most common detection techniques for epigenetic modifications and microRNAs expression used in pathology.

Numerous techniques have been developed to explore the epigenetic processes, not only at gene level but also genome-wide. Those techniques are widely applied to pathology samples, such as formalin-fixed paraffin-embedded (FFPE) tissues or cytology samples, out of which bisulfite modification of DNA and chromatin immunoprecipitation outline the basis for tracking DNA methylation changes and chromatin modifications, respectively. For microRNA expression alterations, quantitative reverse transcription polymerase chain reaction (qRT-PCR) is normally the elected technique (**Figure 2**).

### DNA Methylation in Hürthle Cell Tumors

CpG islands are short stretches of DNA encompassing high GC content and CpG dinucleotides (cytosine 5′ to a guanine, separated by a phosphodiester bond) in comparison with the rest of the genome. CpG islands account for about 1–2% of the genome and are usually located in the 5′ regulatory region of genes. CpG nucleotides are detected in a lower than expected proportion based on the G/C content, and are not homogenously distributed throughout the human genome. CpG islands are regions of human genome rich in GC content that include the promoters of 60% of protein coding genes. In humans, DNA methylation occurs most often in CpG islands, and is frequently localized close to promoters of housekeeping genes and in up to 40% of tissue specific genes (13, 14). In general, methylated CpG islands have been correlated with the transcriptional silencing of the associated genes and non-coding genomic regions, although there are exceptions. Thus, DNA methylation is frequently known as a major repressive epigenetic mark. Nevertheless, DNA methylation function varies with the genomic context (15, 16). If DNA methylation takes place in proximal and distal regulatory elements (i.e., promoters and enhancers, respectively), it represses transcription by affecting the binding of transcription factors and/or recruiting enzymes that modify chromatin structure. However, DNA methylation of the gene body may enhance transcriptional elongation and affect splicing. Therefore, DNA methylation is a key player in the regulation of gene expression, and it has been implicated in many cellular processes, such as imprinting (17, 18), X-chromosome inactivation (19), and the establishment and maintenance of cell type-specific expression programs. In other words, the hypermethylation of the CpG islands in promoter regions, which has been commonly detected in cancer cells, usually leads to the silencing of tumor suppressor genes, whereas the global hypomethylation of DNA has been more frequently reported in association with tumor progression. This pattern of global hypomethylation and regional hypermethylation, might play a pivotal role in human carcinogenesis (20).

DNA methylation is catalysed by a family of DNA methyltransferases (DNMTs) that transfer a methyl group from S-adenyl methionine (SAM) to the fifth carbon of a cytosine residue to form 5mC; DNMT1 maintains the DNA methylation pattern from the parental DNA strand in the newly synthesized daughter and is capable of repairing DNA methylation, whereas DNMT3a and DNMT3b can harbor a new methylation pattern without requiring hemi-methylated DNA, thus naming this process as “*de novo*” DNMT. These two types of methylation patterns are known as “active” and “*de novo*”, respectively, and both contribute to the (im)balance of the roadmap which may lead to tumorigenesis (21). Cancer genome is typically characterized by global hypomethylation concomitantly with hypermethylation of CpG islands in the promoters of genes that play important roles in regulating cell cycle, apoptosis, differentiation, and cell adhesion (9).

Nowadays, DNA methylation has been gaining attraction for its important wellspring of promising cell-type specific cancer biomarkers, especially since there is yearslong protecting stability of DNA methylation, even in formalin-fixed samples, which makes it easily detectable by well-established techniques (**Figure 1**), including in cytology materials (14).

There has been a limited number of DNA methylation studies of TC in the literature, and most of them were focused on PTC. The Cancer Genoma Atlas (TCGA) analysed the methylation profiles of 496 cases of TC using a high-density platform (Illumina Infinium HM 450 array), and suggested a classification based on the genomic profiles, which would support a rational patient disease management (20, 22). However, when it comes to HCN, the literature available is even more limited (**Table 1**). One explanation for this is that these neoplasms were classified as a variant of FTA/FTC and many of the studies were designed before the 4^th^ edition of the WHO Classification of Tumors. Also, it is possible that the fact that no differences on the global methylation of cytosine were found between HA and FTA by Galusca et al. (23) led to a lack of interest in new studies in this field. Galusca et al. designed a study based on immunohistochemistry of benign and malignant thyroid neoplasms including ten Hürthle cell adenomas (HA) (23). The authors concluded that benign tumors (FA or HA and MNG) had a significant higher global DNA methylation level than the studied TC cases (P<0.001) (23). The analyses of benign lesions (hyperplasia, FA and HA) did not find differences between benign lesions and normal thyroid tissues. These results are in line with those previously published concerning 5–methylcytidine level in lung cancers cells (31, 32), breast carcinoma (33) or colorectal adenocarcinomas (34) where no differences where found in relation to the surrounding normal tissues (35).

**Table 1:** Summary of the main DNA methylation and Histone modifications changes reported in Hürthle cell neoplasms (HCN).

	Sample size	Molecular aterations	Methods	Reference
DNA methylation	16 FA,	Lower level of 5-mC immunostaining in TC versus benign tumors or adjacent normal thyroid parenchyma (*P*<0.0001)	Immunohistochemistry	Galusca et al. (23)
	10 HA,			
	19 MNG,			
	17 PTC,			
	6 FTC			
	61 FA,	Hypomethylation of cg10705422, cg17707274, and cg26849382 differentiated nonmalignant (FA, HA, and NIFTP) tumors from differentiated TC	Illumina HumanMethylation EPIC bead array and pyrosequencing	Park et al. (24)
	24 HA,			
	56 NIFTP,			
	120 PTC,			
	27 FTC,			
	5 HCC			
	6 HN,	Increased *KIF1A* probability of methylation in tumor samples (14% in cancer tissue versus 0% in normal and benign; *P* = 0.02)	Quantitative methylation specific PCR (QMSP)	Brait et al. (25)
	12 FA,			
	6 AN,			
	1 AH,	Increased *RASSF1A* methylation levels in cancer versus benign samples samples (*P* = 0.05)		
	6 MNG,			
	1 multinodular hyperplasia,			
	12 HA,	Negative univariate correlation between *BRAF* mutation and *RASSF1A* methylation (*P* = 0.035)		
	27 PTC,			
	7 FTC,			
	2 HCC,	Positive univariate correlation between *BRAF* mutation and methylation of *RARβ2* (*P* = 0.05), *TIMP3* (*P* = 0.05) and *DCC* (*P* = 0.01)		
	8 MTC			
	5 NTS	*CASP8*, *RASSF1* and *NIS* methylated in:		Stephen et al. (26)
	3 hyperfunctioning nodules	- 5/5, 4/5 and 1/5, respectively, in normal thyroid samples	Methylation specific multiplex ligation-dependent probe amplification (MS-MLPA) assay for 24 tumor suppressor genes and methylation-specific PCR (MSP) for NIS gene	
	11 PTC	- 3/3, 2/3 and 1/3, respectively, in hyperthyroid samples		
	1 FTC	- 9/13, 10/13, and 7/13 respectively, in TC		
	1 HCC	- 3/11, 4/11 and 3/11, respectively, TC cases matched with normal thyroid tissue		
		*RASSF1* differentially highly methylated in classic FTC versus HCC (*P*<0.001)		
	27 FTC,	*RASSF1* differentially highly methylated in classic FTC versus normal adjacent tissue (*P*=0.001) Extra-thyroidal extension associated with *DAPK1* (*P*=0.014) and *ESR1* (*P* = 0.036) methylation; late-stage disease associated with methylation of *DAPK1* (*P*= 0.034) and *ESR1* (*P* = 0.035)	Bisulfite Modification and Quantitative MSP	Stephen et al. (27)
	26 HCC			
	50 NTS,	Cluster 1: enriched with FAs, nodular goiters, and minimally invasive FTCs, showing more frequent hypermethylation events; enriched by signal transduction–related genes	Genome-wide DNA methylation assay (450k platform, Illumina) followed by unsupervised hierarchical clustering analysis	Bisarro Dos Reis et al. (28)
	17 benign thyroid lesions,	Cluster 2: all normal thyroid tissue samples		
	60 PTC,	Cluster 3: exclusively PTC samples, with evident methylation loss; enriched by signal transduction–related genes		
	8 FTC,	Cluster 4: all ATC/PDTC, lymphocytic chronic thyroiditis, and remaining FTC/HCC (most extensively, enriched by alterations involved in immune response invasive)		
	2 HCC,			
	1 PDTC,			
	3 ATC			
		Benign thyroid lesions and FTC with greater number of methylated CpG in comparison with normal thyroid tissue; hypomethylation predominant in PTC and PDTC		
		FTC/HCC with hypermethylation in ~3/4 of all identified probes differentially methylated		
		Mutations in 59% of tumors [33/56]:	Whole exome sequencing (WES)	Ganly et al. (29)
	56 HCC	A. Chromatin-modifying complexes		
		- SWI/SNF, ISWI/CHD, and INO80 families - *ARID1A* (4%), *CHD2*, 4, *5*, *8*, *9*, and H (12%) - Histone acetyltransferase (*CREBBP* [5%] and *BRD7* [7%]) - Histone methyltransferases (KMT2C [5%], NSD1 [4%], and EZH1 [4%]) - Histone deacetylases (*HA T7* [2%] and *SIRT6* [2%]) - Histone demethylases (*PHF2* [4%] and *KDM2B*, *4C*, and *5C* [6%]) - Histones (*HIST1H1E* [2%] and *HIST1H3D* [2%])		
		B. DNA modifiers (9% [6/56 tumors]):		
		- *DNMT1* [2%] and *DNMT3A* [2%]) - DNA demethylases (*TET1* [2%] and *TET2* [5%])		
		*ARHI* underexpression in FTC, including six oFTC versus FAs, (*P* = 0.0018)		
	5 NTS,		Real-time quantitative polymerase chain reaction (RT-PCR)	Weber et al. (30)
	14 FA,	*ARHI* underexpression in all minimally invasive FTC (2 classic FTC and 2 HCC)		
	11 FTC,			
	6 HCC,			
	7 PTC	*ARHI* underexpression in FTC versus normal thyroid tissue (*P* = 0.022) or FAs (*P* = 0.0091)	Sodium bisulfite genomic sequencing	
		*ARHI* underexpression in FTC versus PTC (*P* = 0.022)	Quantitative PCR (qPCR)	
Histone modifications	56 HCC	Mutations in histone acetyltransferases (*CREBBP* [5%] and *BRD7* [7%]), histone methyltransferases (*KMT2C* [5%], *NSD1* [4%], and *EZH1* [4%]), histone deacetylases (*HAT7* [2%] and *SIRT6* [2%]), histone demethylases (*PHF2* [4%] and *KDM2B*, *4C*, and *5C* [6%]), and histones (*HIST1H1E* [2%] and *HIST1H3D* [2%])	Whole exome sequencing (WES)	Ganly et al. (29)

FA, follicular adenoma; HA, Hürthle cell adenoma; MNG, multinodular goiter; PTC, papillary thyroid carcinoma; FTC, follicular thyroid carcinoma; NIFTP, non-invasive follicular thyroid neoplasm with papillary-like nuclear features; HCC, Hürthle cell carcinoma; HN, hyperplastic nodule; NTS, normal thyroid specimens; AH, adenomatoid hyperplasia; MTC, medullary thyroid carcinoma; PDTC, poorly differentiated thyroid carcinoma; ATC, anaplastic thyroid carcinoma; oFTC, oncocytic variant of follicular thyroid carcinoma.

One of the recent studies by Park et al. (24), which was designed after the 4^th^ edition of the WHO Classification of Tumors (3), quantitatively profiled the genome-wide DNA methylation in a group of thyroid tumors by means of the Illumina HumanMethylation EPIC bead array. Using a cohort encompassing 24 HA and 5 HCC alongside with PTC, FTC and non-invasive follicular thyroid neoplasm with papillary-like nuclear features (NIFTP) cases, the authors verified a statistically significant hypomethylation of three specific marks - cg10705422, cg17707274, and cg26849382 - in malignant tumors. They also found that in this tumor group the three marks were significantly associated with recurrent or persistent disease. The authors concluded that DNA methylation levels of those three markers could be a promising novel diagnostic/prognostic biomarker for well-differentiated thyroid cancer (WDTC). Considering that alterations in DNA methylation play a role in tumorigenesis and disease progression, these DNA methylation markers may be clinically useful for stratifying thyroid tumors in a more efficient way (24).

Brait et al. (25) also evaluated *BRAF* mutation and promoter DNA methylation for 22 selected cancer-related genes, in a group of thyroid malignant and benign cases, including 12 HA and 2 HCC, in comparison with adjacent normal thyroid. The relevance of the upregulation of the RAF/MEK/MAPK kinase pathway in TC, either by a *BRAF* activating mutation or *RASSF1A* methylation (hypermethylation mutually exclusive with *BRAF* mutation) silencing, was further confirmed by this study, without any difference between Hürthle and non-Hürthle morphology. Previously, hypermethylation of tumor supresor gene *RASSF1A* was reported in 75% (9 of 12) of FTCs, as well as in a smaller percentage of benign adenomas (44%), and PTCs (20%) (36), indicating that this may represent an early step in follicular cell-derived thyroid tumorigenesis. Following this hypothesis, Stephen et al. (26) examined the promoter hypermethylation of 24 tumor suppressor genes using the methylation-specific multiplex ligation-dependent probe amplification (MS-MLPA) assay, as well as that of Na-I symporter (NIS) gene using methylation-specific PCR (MSP). In a pilot study of 21 thyroid cases, including normal thyroid tissue and benign thyroid lesions, PTC and anaplastic thyroid carcinoma (ATC), 2 cases of FTC (one of them formarly named as an oncocytic variant of FTC, now known as HCC). They have concluded that *RASSF1*, *CASP8* and *NIS* were frequently methylated in their promoters, which may indicate a role in early tumorigenesis, regardless of the cell type (26).

In 2015, again Stephen et al. (27) analysed retrospectively the promoter methylation status in a cohort of 26 HCC and 27 FTCnbsp;cases, both in comparison with each other and with their adjacent normal tissues. The group found that *RASSF1* demonstrates a significant differential methylation between FTC-Classic and FTC-Hurthle, with higher methylation levels in FTC-Classic (*P <*0.001), suggesting its use as a molecular marker to differentiate these two subtypes. Comparisons with their matching adjacent normal tissues were also significant (*P*=0.01) (26).

Bisarro Dos Reis et al. (28) performed an unsupervised clustering analysis of the genome-wide DNA methylation profiles in a series of thyroid tumors. Although HCCs were not separated from FTCs, the FTC/HCC group clustered together with the ATC/poorly differentiated thyroid carcinoma (PDTC) group and with lymphocytic thyroiditis. This cluster showed major alterations in genes related to immune response. Of note, the FTC/HCC group showed hypermethylation in approximately 3/4 of all identified probes differentially methylated (28).

Ganly et al. (29) recently found that mutations in genes encoding for chromatin and DNA modifying enzymes are frequent, with 59% of the 56 studied HCC cases harbouring such mutations.

Finally, it is noteworthy the contribution of Weber et al. (30) for the understanding of the carcinogenesis of FTC when they showed that the tumor suppressor gene aplaysia ras homolog I (*ARHI*) was frequently underexpressed in FTCs, even in minimally invasive tumors, including 6 oncocytic variants (*P*=0.0018), as compared to PTC or FA. Based on their their studies, the authors have suggested that silencing of the putative maternally imprinted tumor suppressor gene *ARHI*, primarily by large genomic deletion associated with hypermethylation of the genomically imprinted allele, is an important event in follicular thyroid carcinogenesis.

### Histone Modification in Hürthle Cell Tumors

DNA is associated with histone proteins to form a condensed structure known as chromatin. Chromatin encompasses 147 bp of DNA bundling up an octamer of four histone proteins (H2A, H2B, H3, and H4) (37). Histone modifications can go through various types of post-translational changes, including methylation, acetylation, phosphorylation and ubiquitination, and these may modulate gene silencing concomitantly with DNA promoter methylation (17, 38). In general, acetylation of histones, such as H3 and H4, and methylation of the lysine-4 residue of histone H3 (H3K4) are associated with transcriptional activation and active genes (39, 40). Deacetylation, on the other hand, is associated with gene silencing of tumor suppressor genes in the carcinogenesis process (40, 41). Methylated DNA recruits methylbinding proteins (MBDPs), which have methyl-CpG-binding domains (MBD), to hypermethylated DNA. MBDPs associate with histone deacetylases, resulting in chromatin remodeling and gene silencing. In addition to these mechanisms of silencing, histone methyltransferase (HMTs) repress transcription by methylation of lysine 9 of histone 3 (H3K9) or lysine 27 of histone 3 (H3K27) (40). Fewer studies have been published on the histone modifications in thyroid neoplasms (**Table 1**). Puppin et al. (42) reported changes in the overall histone acetylation levels in TC, with lower levels of acetylated H3 at K18 residue in undifferentiated cancers in comparison with differentiated types of cancers. Of interest, based on a study that was carried out on the human TC-derived cell lines TPC-1, KTC-1, NPA, WRO, ARO, DRO, 8505C, 8303C, the CpG promoter region of the thyroid transcription factor-1 (*TTF-1*), an essential gene for thyroid organogenesis governing thyroid functions by regulating the expression of thyroid-specific genes including thyroglobulin (*TG*), thyroid peroxidase (*TPO*), thyroid-stimulating hormone receptor (*TSHR*) and *NIS*, was found to be hypermethylated in those differentiated and undifferentiated TC cell lines, along with a reduction in acetyl-H3-K9 and an increase in dimethyl-H3-K9 in cells seen in a subset that lost *TTF-1* expression (43). As mentioned before, epigenetic modification mutations have been identified as common events in genes of HCC encoding chromatin or DNA modifiers, but mutations were also detected in histone acetyltransferases (*CREBBP* [5%] and *BRD7* [7%]), histone methyltransferases (*KMT2C* [5%], *NSD1* [4%], and *EZH1* [4%]), histone deacetylases (*HAT7* [2%] and *SIRT6* [2%]), histone demethylases (*PHF2* [4%] and *KDM2B*, *4C*, and *5C* [6%]), and histones (*HIST1H1E* [2%] and *HIST1H3D* [2%]) (29).

### RNA-Associated Silencing in Hürthle Cell Tumors

MicroRNAs (mi-RNAs) are small (19–23, 31, 32) non-coding RNAs, which control gene expression through post-transcriptional regulation of various cellular processes, such as differentiation, proliferation and apoptosis (44). Signatures associated with their expression have been related to diagnosis, staging, prognosis, and response to treatment in human tumors (45–47). Changes in mi-RNA expression are involved in carcinogenesis and tumor progression, through downregulation of tumor suppressor genes and/or upregulation of oncogene (48–51). One of the first pivotal series which characterized the mi-RNA expression profile of different benign and malignant thyroid neoplasms, including HA and HCC, was published by Nikiforov et al. (52). The ten most up-regulated miRNAs in HA when compared with normal thyroid tissue were, respectively from higher to lower, miR-31, miR-339, miR-183, miR-182, miR-181b, miR-221, miR-96, miR-182, miR-224 and miR-203. In comparison with thyroid hyperplastic tissue, miR-31, miR-339, miR-183, miR-221 and miR-203 were significantly more expressed in HA. In HCC, the ten most up-regulated miRNAs were miR-187, miR-221, miR-339, miR-183, miR-222, miR-181b, miR-182, miR-213, miR-96, miR-197, respectively from higher to lower levels. miR-187, miR-221, miR-339, miR-183, miR-222, and the miR-197 were significantly more expressed when compared with hyperplastic thyroid tissue. Interestingly, within the adenoma group, non-oncocytic tumors expressed a pattern of miRNA different from that of HA. Indeed, miR-200a was the most expressed miRNA in HA and miR-31 was the most expressed in HCC. A cluster analysis showed that clusters of miRNA expression in HCN are different from those of non-oncocytic tumors, which supports the theory that HCN represent a unique class of thyroid tumors, as opposed to a subgroup of follicular-derived tumors (2). The evidence that a cluster of identical miRNAs is present in both benign and malignant follicular tumors with Hürthle cell characteristics supports the assumption that these represent a phenotype that is superimposed on different oncogenic genotypes (53). Vriens et al. (54) found that four of ten miRNAs, initially mapped by a miRNA array, were significantly and differentially expressed when comparing benign and malignant thyroid neoplasms by real-time quantitative polymerase chain reaction (RT-qPCR). In particular, miR-100, miR-125b, miR-138, and miR-768-3p were found to be overexpressed in malignant follicular tumors and in HCC samples when compared with HA (54). The accuracy for distinguishing benign from malignant HCN was 98% (miR-138 and miR-768-3p) (54). Dettmer et al. (55) assessed the difference in miRNA expression between PDTC and WDPTC using PCR-Microarrays. In this study, they found that the oncocytic variant of PDTC (oPDTC) presented an upregulation of miR-221 and miR-885-5p when compared with the non-oPDTC (55). When oPDTC were compared with HCC, there was a loss of expression of miR-125a-5p, -183-3p, -219-5p, -221 and miR-885-5p. In addition, miR-222 was twice more expressed in oPDTC compared with HCC (55). Petric et al. (56) assessed whether there was any miRNA which could be predictive of metastases in HCC, based on a series of 39 patients, and using TaqMan miRNA assays targeting six miRNAs (miR-138, miR-183, miR-221, miR-222, miR-768-3p, and miR-885-5p). These authors found that miR-138 and miR-768-3p were significantly downregulated in HCC tumor samples, as well as in patients with metastatic disease, but not in patients with non-metastatic disease, in comparison with normal tissue (56). In tumors of patients without metastases, miR-221 and miR-885-5p were significantly upregulated when compared with normal tissue. When the difference of miRNA expression in tumor versus normal tissue was compared between patients with and without metastases, miR-183, miR-221, and miR885-5p were significantly downregulated in patients with metastases, suggesting that the expression of miR-183, miR-221, and miR-885-5p in tumor tissue is inversely correlated with the risk of distant metastases in patients with HCC (56). A study by Covach et al. (57), which assessed metastases associated lung adenocarcinoma transcript 1 (MALAT1) and miR-RNA-885-5p through *in situ* hybridization (ISH) found that MALAT1 was more highly expressed in HCCs compared with FTCs, although the differences were not statistically significant (*P*= 0.06). Likewise, miR-885 expressed in similar levels in FTCs and HCCs. Jacques et al. (58) reported the results of transcriptomic analysis, which led to the identification of 13 genes allowing the discrimination between thyroid adenomas, HA/HCC and PTC. The overall miRNA analysis revealed 10 differentially expressed miRNAs, whose levels were confirmed by qPCR (58). In their study, *TP53* and *RUNX1* were the main genes regulated by the selected miRNAs, which proved to play a role in thyroid tumor development and differentiation. The targeted assay tested by these authors allowed the specific investigation of thyroid mitochondrial metabolism and tumorigenesis genes, which are partially regulated by miRNAs. In this study, mitochondrial metabolism of HA/HCC appeared to differ from that of PTC, suggesting metabolic variations of the organelle in these two tumor types (58) (**Table 2**).

**Table 2:** Summary of the main RNA-associated silencing changes reported in Hurthle cell neoplasms.

	Sample size	Molecular aterations	Methods	Reference
RNA-associated silencing	5 NTS,	Upregulation of miR-31, miR-339, miR-183, miR-182, miR-181b, miR-221, miR-96, miR-182, miR-224 and miR-203 in HA versus NTS	Real-time quantitative polymerase chain reaction (RT-qPCR)	Nikiforov et al. (52)
	5 HN			
	23 PTC,			
	9 FTC or HCC,	Upregulation of miR-31, miR-339, miR-183, miR-221 and miR-203 in HA versus hyperplastic tissue		
	8 FA or HA,			
	4 ATC,			
	4 PDTC,	Upregulation of miR-187, miR-221, miR-339, miR-183, miR-222, miR-181b, miR-182, miR-213, miR-96, miR-197 in HCC		
	2 MTC			
		Upregulation of miR-187, miR-221, miR-339, miR-183, miR-222, miR-197 in HCC versus hyperplastic tissue		
		Overexpression of miR-100, miR-125b, miR-138 and miR-768-3p in FTC and HCC versus HA		
	39 HCC (22 non-metastatic and 17 with regional or distant metastases)	miR-138 and miR-768-3p downregulated in HCC compared with normal tissue (*P* = 0.013 and *P* = 0.010, respectively) and in tumors with metastases versus non-metastatic disease (*P* = 0.030 and *P* = 0.048, respectively)	TaqMan MicroRNA Reverse Transcription Kit and specific TaqMan miRNA assays	Petric et al. (56)
		miR-221 and miR-885-5p upregulated in tumors versus normal tissue (*P* = 0.042 and *P* = 0.027, respectively)		
		miR-221 and miR-885-5p significantly upregulated in tumors without metastases versus normal tissue (*P* = 0.019 and *P* = 0.024)		
		miR-183, miR-221, and miR885-5p significantly downregulated in HCC with metastases (*P* = 0.027, *P* = 0.019 and *P* = 0.024, respectively)		
	7 NTS,	miR-100, miR-125b, miR-138 and miR-768-3p overexpressed in FTC versus FA	TaqMan miRNA assay was miRNA array and RT-qPCR	Vriens et al. (54)
	14 HN,	(*P* < 0.001) and in HCC versus HA (*P* < 0.01)		
	15 FA,			
	20 PTC,	Only miR-125b overexpressed in FTC (*P* < 0.05) versus FA		
	12 FTC,			
	12 HA,	miR-768-3p overexpressed in FVPTC and benign samples (*P <*0.001)		
	20 HCC,			
	4 ATC,	miR-138 overexpressed in FNA samples (*P* = 0.04) which were malignant versus benign		
	125 indeterminate (cytolology)			
	8 NTS,	Upregulation of miR-221 and miR-885-5p in oncocytic versus non-oPDTC	PCR-Microarrays	Dettmer et al. (55)
	14 PDTC,			
	13 oPDTC,	Downregulation of miR-125a-5p, -183-3p, -219-5p, -221 and miR-885-5p in oPDTC versus HCC		
	72 WDTC			
		Upregulation of miR-222 in oPDTC versus HCC		
	12 NTS,	*MALAT1* highly expressed in HCCs compared with FTC (n.s.)	In situ hybridization (ISH) and RT-qPCR	Covach et al. (57)
	25 FA,	miR-885 expressed in similar levels in FTCs and HCC		
	25 HA,			
	25 FTC,			
	24 HCC			
	53 NTS,	13 genes with discriminatory capacity between tumors:	cDNA and miRNA microarrays and RT-qPCR	Jacques et al. (58)
	10 WDT-UMP	- *TP53*, *HOXA9*, *RUNX1*, *MYD88*, and *CITED1* are implicated in tumorigenesis - *MRPL14*, *MRPS2*, *MRPS28*, and *COX6A1* are involved in mitochondrial metabolism - *CaMKIINalpha* and *TPO* are involved in thyroid metabolic pathways - *TPO* (FA), *COX6A1*, (HCC), *MRPL14*, *CITED1*, and *CaMKIINalpha* (PTC) significantly overexpressed in tumors versus normal tissue - *HOXA9* under expressed in FAs and PTCs - *MRPS2*, *MRPS28*, and *HOXA9* upregulation in HCC versus FA and PTC		
	25 FA,			
	38 oFTC,			
	19 PTC			

NTS, normal thyroid specimens; HN, hyperplastic nodule; PTC, papillary thyroid carcinoma; FTC, follicular thyroid carcinoma; HCC, Hürthle cell carcinoma; FA, follicular adenoma; HA, Hürthle cell adenoma; ATC, anaplastic thyroid carcinoma; PDTC, poorly differentiated thyroid carcinoma; MTC, medullary thyroid carcinoma; MNG, multinodular goiter; NIFTP, non-invasive follicular thyroid neoplasm with papillary-like nuclear features; oPDTC, oncocytic poorly differentiated thyroid carcinoma; WDT-UMP, well-differentiated tumor of uncertain malignant potential; oFTC, oncocytic variant of follicular thyroid carcinoma.

### Hürthle Cell Tumors and Epigenetic Therapy

Previous studies have suggested that patients with HCC should be treated as non-HCC patients with equivalent tumor staging (59). Specific recommendations for the treatment of HCC are lacking (60), and the management of HCC tends to be similar to that of FTC, acknowledging two particular characteristics: locoregional lymph node and distant metastases are more frequent, and metastatic HCC tend to be less prone to radioiodine-avidity. In general, the HCC clinical management follows the most recent ATA guidelines and National Comprehensive Cancer Network (NCCN) Thyroid Cancer guidelines (61, 62). Treatment options for recurrent HCC include radioiodine, radiofrequency ablation, ethanol ablation, external beam radiotherapy, and systemic treatment. Systemic treatment should be considered for patients with radiodine-refractory unresectable persistent, recurrent or metastatic disease. The options include tyrosine kinase inhibitors (TKIs), such as levantinib and sorafenib, but despite the benefits shown in progression-free survival, they have not shown any improvements in overall survival (63, 64).

The promise of epigenetic alterations as therapeutic targets is that, unlike mutations, they can be potentially reverted. Azacitidine and decitabineare cytidine analogues, which inhibit DNA methyltransferase, were first synthesized in the early 1960s and were approved for the treatment of myelodysplastic syndromes. While their demethylating effect depends on incorporation of derived deoxy-azanucleotides into DNA, azacitidine-induced cytotoxicity is mainly due to incorporation into RNA in the cell cycle phase G1 at low drug concentrations, and to the incorporation into both RNA and DNA in the G1 and S phases of the cell cycle (65). Decitabine was shown to cause hypomethylation of DNA and intra-S-phase arrest of DNA replication, as its activity is exclusive to the incorporation into DNA of replicating cells (66). A phase 1 trial is currently assessing the ability of azacitidine to restore iodine uptake by dedifferentiated FTC and PTC, enabling detection and treatment with radioiodine in patients with metastatic disease, and will determine the efficacy of azacitidine in combination with radioiodine in this patient population. Interestingly, decitabine was shown to revert the *TSH-R* and *NIS* expression in human TC cell lines (67–69), having an inhibitory effect on the growth of undifferentiated TC cells (70). A phase 2 clinical trial assessing the effect of decitabine in patients with stage IV PTC and FTC unresponsive to radioiodine has completed recruitment and results are pending publication.

HDAC inhibitors have been studied in various tumor types and have shown promising results, particularly in hematological malignancies (71). Previous studies have showed that HDAC inhibitors could induce apoptosis and cell cycle arrest in ATC cell lines (72), and that these effects would likely be linked to an increase in the transcription of *TP53*, as well as to the modulation of cell cycle-related molecules (73–75). In addition, HDAC inhibitors, including depsipeptide and trichostatin, have effects on the reexpression of NIS, TPO and Tg genes, with an increased radioiodine uptake in PDTC and ATC cell lines, *in vitro* and *in vivo* (76, 77). The TSHR and NIS are key players in radioiodine-based treatment of differentiated thyroid cancers. Agostino et al. (78) performed the analysis of NIS and TSHR genes expression, and of the epigenetic control occurring at the gene promoter level after inhibition of RAS–BRAF–MAPK and PI3K–Akt–mTOR pathways in four human TC cell lines. The authors found that, in TC cells, MEK and Akt signaling pathways control pos-/translational modifications of histones at specific genes, and these changes are noted even before the effects become more obvious in the proliferation rates. Recently, Wächter et al. (79) studied the cytotoxic effects of histone deacetylase inhibitors panobinostat, vorinostat (suberoylanilide hydroxamic acid (SAHA)), and trichostatin A in five TC cell lines, including PTC, FTC and ATC. All three compounds showed cytotoxic effect and a strong re-expression/over-expression of *NIS* transcript independently of the cell line. This recapitulates part of the results mentioned previously for poorly differentiated and anaplastic tumors (77), and supports the idea that the cell lines expressing low or absent NIS could be the most sensitive to deacetylase inhibitors. NIS expression could be modulated by the expression of miRNAs belonging to Let7 family (80). In the Watcther et al. study (79), treatment with these deacetylase inhibitors led to a stable expression or upregulation of H19, a long non-coding RNA highly expressed during tumorsigenesis in several tumors, including TC (81), in the tested TC cell lines. Interestingly, H19 had been shown to inhibit Insulin Receptor Substrate 1 (IRS-1), reducing cell viability, migration, and invasion in TC (82), which supports the thesis that H19 may have a tumor suppressor role in TC cells treated with deacetylase inhibitors, leading to differentiation and cell death (79). Finally, deacetylase inhibitors also led to the down-regulation of *TTF1* transcripts, a thyroid transcription factor with oncogenic properties, in three cell lines. All these are relevant characteristics of deacetylase inhibitors for which clinical utility in patients with radioiodine refractory TC must be tested in clinical trials. Currently, there are phase 1 and 2 clinical trials using HDAC inhibitors such as romidepsin, belinostat, panobinostat and valproic acid in patients with TC, including limited cases of HCC, but the results are not encouraging (83–87).

Finally, although it is becoming evident that mi-RNAs play a role in the HCC pathogenesis, with an opportunity for a future as diagnose markers, there are still many unanswered questions on how they can contribute to the selective methylation or demethylation of TC genes. In what concerns treatment application, mi-RNAs are still far from being a druggable target, although it is possible that an acceleration of this pathway may be seen in the near future as RNA-based therapies are becoming an area of high investment, such is the case of the recent advances in the prevention area in vaccines. In vitro experiments by Vriens et al. (54), using a FTC cell line with overexpression of miR-100, miR-125b, and miR-138, found that the inhibition of the miRNAs by their targeted anti-mir led to cell growth arrest. On the other hand, the overexpression by pre-miRNAs (pre–miR-100, pre–miR-125b, and pre– miR-138) dramatically increased proliferation. This, and other pre-clinical reports, may pave the way for the next step into clinical trials, which has not yet started. As HCC are known for their mitochondrial DNA mutations and respiratory complex(es) metabolic disfunction, it would be very interesting to explore the potential treatment opportunities oppened by the fact that miRNAs are translocated into mitochondria - thus modifying the expression of the mitochondrial genome. Likewise, the alterations in the mitochondrial DNA, which also contribute to epigenetic modifications of the nuclear DNA (88), could, at least in theory, be tackled in HCC.

## Conclusions

Since Waddington’s introduction of epigenetics in the 1940s, there has been a significant development in the field and its application to cancer. The extension of these advances holds promises in establishing novel diagnostic and prognostic markers, with patient management implications, if not at the level of readily available systemic treatment options, certainly in the surgical and follow-up standards.

However, research on thyroid neoplasms has remained relatively limited by comparison with other cancers. One such case is the HCN, which was endorsed by the 4^th^ edition of the WHO Classification of Tumors. As one can see in this review, the evidence pertaining to the epigenomics of HCN is poor and inconsistent. In addition, a limitation in our review is that different referenced studies have used different morphological criteria to diagnose tumors falling into the spectrum of oncocytic follicular epithelial derived neoplasms, which are not necessarily follicular patterned-neoplasms, and therefore some of the epigenomics evidence trends may not always reflect a HCN hystotype. This reinforces the need for determined and focused efforts in research and publication on the topic, taking into consideration the new pathological classification of HCN. Future advances in the research will allow the definition of diagnostic markers as well as specific guidelines towards a more personalized and effective treatment of HCN.

## Author Contributions

Conceptualization: SC, AL, VM. Writing – original draft: SC, AL, MP. Writing – review and editing: SC, AL, PS, VM. Supervising: VM. All authors contributed to the article and approved the submitted version.

## Funding

This work was financed by FEDER—Fundo Europeu de Desenvolvimento Regional funds through the COMPETE 2020—Operacional Programme for Competitiveness and Internationalization (POCI), Portugal 2020, and by Portuguese funds through FCT - Fundação para a Ciência e a Tecnologia/Ministério da Ciência, Tecnologia e Inovação in the framework of the project “Institute for Research and Innovation in Health Sciences” (POCI-01-0145-FEDER-007274). Additional funding by the European Regional Development Fund (ERDF) through the Operational Programme for Competitiveness and Internationalization—COMPETE2020, and Portuguese national funds *via* FCT, under project POCI-01-0145-FEDER-016390: CANCEL STEM and from the FCT under the project POCI-01-0145-FEDER-031438: The other faces of telomerase: Looking beyond tumor immortalization (PDTC/MED_ONC/31438/2017). Additional funding through the Sociedade Portuguesa de Endocrinologia, Diabetes e Metabolismo – Bolsa Edward Limbert MERCK/SPEDM 2019. SC is supported by FCT in the framework of a PhD grant (SFRH/BD/147650/2019).

## Conflict of Interest

The authors declare that the research was conducted in the absence of any commercial or financial relationships that could be construed as a potential conflict of interest.
